# Immunomodulatory properties of high mobility group box 1 and its potential role in brain injury: Review article

**DOI:** 10.1016/j.amsu.2020.09.025

**Published:** 2020-09-20

**Authors:** Thomas Tommy, Andi A. Islam, Mochammad Hatta, Agussalim Bukhari

**Affiliations:** a Department of Neurosurgery, Faculty of Medicine, Universitas Pelita Harapan, Tangerang, Indonesia; b Department of Neurosurgery, Faculty of Medicine, Universitas Hasanuddin, Makassar, Indonesia; c Molecular Biology and Immunology Laboratory, Faculty of Medicine, Universitas Hasanuddin, Makassar, Indonesia; d Department of Nutritional Science, Faculty of Medicine, Universitas Hasanuddin, Makassar, Indonesia

**Keywords:** HMGB1, Brain injury, Inflammation

## Abstract

**Background:**

Human mobility group box 1 (HMGB1) is a novel biomolecular agent which has a major part in inflammation process. HMGB1 has been known to be a strong pro-inflammatory factor as damage associated molecular pattern (DAMP) which its interaction with its receptor, the receptor of advanced glycation end products (RAGE), will cause positive amplification of inflammation signalling pathway.

Brain injury is one of the major contributors for disability and death which neuroinflammation has a major role in its pathogenesis and influencing its outcome. In neuroinflammation, it has been described that HMGB1 may have a pivotal role in the process.

**Objective:**

The objective of this article is to review the role HMGB1 in brain injury and its immunomodulatory properties.

**Methods:**

A comprehensive search of literature was conducted in PubMed (NIH), Scopus, EMBASE, and Google Scholar database using keyword combinations of the medical subject headings (MeSH) of “HMGB1” and “Brain Injury” and relevant reference lists were also manually searched. All relevant articles of any study design published from year 1990 till June 2020, were included and narratively discussed in this review.

**Results:**

Twenty-four articles were shortlisted and reviewed in this article. Through these articles, we synthesis information on the function and metabolism of HMGB1, immunomodulatory effect of HMGB1, clinical findings and other potential treatment involving HMGB1, and role of HMGB1 protein in brain injury.

**Conclusion:**

HMGB1 has a strong pro-inflammation property which predominantly acts through RAGE pathways.Review registration number reviewregistry966 in www.researchregistry.com.

## Highlights

• HMGB1 has a strong pro-inflammation property which predominantly acts through RAGE pathways.
• This pro-inflammatory process needs to be balanced with anti-inflammatory agents for homeostasis.
• Further studies are needed to support anti HMGB1 therapy in inflammation process.

## 1 Introduction

Human mobility group box 1 (HMGB1) is identified in the 1970s [1] and is discovered in 1999 [2] and has been described as protein in the family of damage associated molecular pattern (DAMP). HMGB1 once was described as sulfoglucoronyl carbohydrate binding protein, amphoterin, and high mobility group protein 1. This protein is an intranuclear non-histone protein with a molecular weight of 30-kDa and is a mediator of inflammation and inflammatory responses following tissue damage (injury or infection) hence it is considered an important late inflammatory mediator. Several studies revealed that HMGB1 has molecular impact in Pathogen-Associated Molecular Patterns (PAMPs) in infection and Damage-Associated Molecular Patterns (DAMPs) in injury [3]. Previous studies showed that HMGB1 has involved in molecular mechanisms of systemic lidocaine treatment for closed fracture musculoskeletal injury in animal model [4,5].

Traumatic brain injury (TBI) is considered as the silent epidemic because of its contribution to worldwide death and disability, with estimated cases of sixty-nine million suffering traumatic brain injury from all causes [6]. The management of TBI cases proves to be high cost and also, with high frequency of disability outcome, will affect the productivity of patients. Inflammation after TBI is now has been recognized as complex interaction between central and peripheral cellular and soluble components. Previous study revealed that CAPE can reduce Neutrophil serum levels there by preventing brain damage in TBI [7].

Any treatment management directed to modulate inflammation in TBI may give better outcome.

## 2 Method

A comprehensive search of literature was conducted in PubMed (NIH), Scopus, EMBASE, and Google Scholar database using keyword combinations of the medical subject headings (MeSH) of “HMGB1” and “Brain Injury” and relevant reference lists were also manually searched. The review was registered in www.researchregistry.com with unique identifying number: reviewregistry966 and Preferred Reporting Items for Systematic Reviews and Meta-Analyses Protocols (PRISMA) criteria also has been followed [8]. All relevant articles, with English language, of any study design published from year 1990 till June 2020, were included and narratively discussed.

## 3 Results

The literature search identified 302 articles. Of them, 24 articles were included in this review.

## 4 Discussion

### 4.1 Function and metabolism of HMGB1

**Fig. 1:**
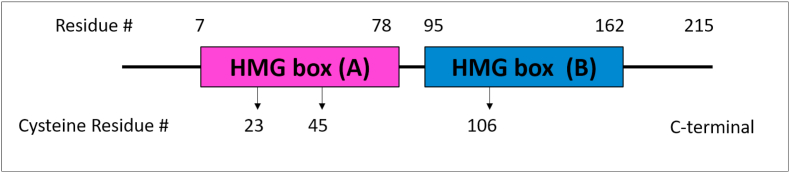
HMGB1 protein structure [11].

HMGB1 gene has a chromosomal location of 13q12.3. HMGB1 protein contains 215 amino acids with two folded DNA binding motifs called box A (9–79 aa) and box B (95–163 aa) and an acidic C tail (186–215 aa). Has two nuclear localization sequences (NLSs) located in box A (24–44 aa) and between box B and C tail (179–185 aa). The protein structure of HMGB1 is depicted on Fig. 1. The biological properties of HMGB1 depend on the redox state of its three cysteine residues (C23, C45, and C106) and will be discussed later on this article [9]. It has been described that HMGB1 box B has been mapped as a pro-inflammatory domain [1] and box A contains binding sites for HMGB1 receptors [10].

### 4.2 Function based on location, binding partners, and redox states

Based on its location, HMGB1 has the following functions; In the **nucleus:** HMGB1 has roles in organizing DNA and nucleosomes and regulating gene transcription (Transcription factor, transcription enhancer, nucleosome sliding, DNA repair, V(D)J recombination, telomere homeostasis) [11]. In **cytoplasm**: involved in inlammasome activation and pyroptosis, regulation of autophagy and apoptosis balance. **Extracellular**: cytokine, chemokine, neuroimmune, and metabolic activities. HMGB1 can be released into extracellular space by two ways, passively or actively. Passive release occurs instantaneously after cell injury or death. Active release of HMGB1 is being done by translocation of nuclear HMGB1 to cytoplasm, requiring the JAK-STAT signalling pathway, and gradual induction of pyroptosis (requires caspase-1 from inflammasome activity) that allows cytoplasmic HMGB1 release into extracellular space. Alternatively, HMGB1 can be delivered to extracellular space by lysosome (exocytosis) [9].

Eleven different HMGB1 receptors have been described. They were TLR/MD-2, RAGE, CD24, Integrin, TIM3, TLR4, TLR2, TLR3, IL-1R1, and CXCR4. The effects of binding of HMGB1 with the respective receptors are described on table.

Exposure of monocytes to HMGB1 stimulated release of TNF, interleukin-1 (IL-1), IL-6, IL-8, and macrophage inflammatory protein (MIP)-1 [9]. It has been demonstrated that HMGB1 is involved in DNA-containing complex mediated immune responses via TLR9 [12] and stimulates the transcription of type-1 interferons, IL-6 and RANTES (Regulated on Activation, Normal T Expressed and Secreted) protein from immune cells or embryonic fibroblasts [13]. Extracellular HMGB1 binds to RAGE and get internalized by endocytosis into intracellular space playing essential role in innate immune responses (modulates interferon and cytokine responses) and also capable of modulating the adaptive (acquired) immune response [9,11]. The summary of HMGB1 receptors has been summarized on Table 1.

**Table 1:** HMGB1 receptors [9].

Receptors	Effects	Complex with
TLR/MD-2	Cytokine release	–
RAGE	Cell migration, pyroptosis, internalization of HMGB1 partner molecules	–
CD24/Siglet10	Anti-inflammatory	–
Integrin/Mac1	Cell recruitment	–
TIM3	Reduced tumor immunity	–
TLR4/MD-2	Cytokine release, increased neuroinflammation	LPS, NMDAR
TLR2	Cytokine release, enhanced autoantibody formation	Pam_3_CSK_4_, nucleosome
TLR3/7/9	Cytokine release	Nucleic acids
IL-1R1	Cytokine release	Il-1α/β
CXCR4	Chemotaxis	CXCL12

HMGB1 can be identified having different redox state (reduced, disulfide bond, and oxidized). Fully reduced HMGB1, or could be said as all-thiols residues, exerts chemotactic activity by forming a hetero complex with CXCL12, which when binds to its receptor, CXCR4, will initiate chemotaxis. In the form of disulfide bond (C23 and C45) and C106 in thiol will give cytokine stimulating activity. The all-thiol HMGB-1 cannot activate TLR4/MD-2 signalling pathway nor disulfide bond HMGB-1 cannot activate the CXCL12/CXCR4 signalling pathway. The fully oxidized form, so called sulfonyl HMGB1, has no identified activity [9].

### 4.3 Immunomodulatory effect of HMGB1

In inflammation process there are two major activity, proinflammation and antiinflammation. Inflammation is a double-edged sword in homeostasis. Even though inflammation process is needed for survival, if not in check, the process would turn up uncontrolled and gives poor outcome. In acute setting (initial insult of injury), cells will passively and actively transport HMGB1 into cell surface or extracellular space, which leads to recruitment of other immune cells (neutrophils) and amplification of the initial activation signal. This initial activation signal is also influenced by other extracellular factors, such as: acute phase proteins (fetuin A, C-reactive protein (CRP), serum amyloid A (SAA)), pentraxin3, complement factors, endogenous glucocorticoids, and cytokines or chemokines [11]. After the initial activation signals, pattern recognition receptors (TLRs [12], RAGE) are activated by their interaction with PAMPs and DAMPs (DNA, dsRNA, LPS, flagellin, peptidoglycan, S100 proteins, heat shock proteins, uric acid, ROS). HMGB1 has been identified could propagate inflammatory process by inducing synthesis of CRP, TNFα, IL-6, and MIP1α and β in human monocytes [14] and modulating the adaptive immune response.

In response of acute inflammation, an anti-inflammatory process is also triggered. DAMPs and PAMPs must be cleared and activated cells need to be returned into resting state. This anti-inflammatory process kept the homeostasis in balance (anti-inflammation and pro-inflammation). Few main anti-inflammatory pathways have been identified: induction of inhibitory miRNAs, SOCS proteins, activation of hypothalamic pituitary axis leading to adrenal corticosteroid production, and stimulation of cholinergic anti-inflammatory pathway. miRNAs, SOCS, and CAP have been identified having effects regulating HMGB1 [11,[15], [16], [17]].

### 4.4 Clinical findings and other potential treatment involving HMGB1

HMGB1 is present in haemorrhagic shock [18,19] because haemorrhagic shock stimulated a systemic release of HMGB1 [20]. In an in vivo model, treatment of neutralizing *anti*-HMGB1 antibody can improve survival and ameliorate gut barrier dysfunction in relevant haemorrhagic shock model [21]. In accordance with this findings, haemorrhagic shock could cause a decrease blood flow to the brain and heart causing myocardial and cerebral ischemia thus releasing HMGB1 [18]. In ischemic stroke, increased concentration of HMGB1 in circulation happened within hours after tissue damage [18].

Intravenously administered lidocaine can be used for transcription inhibition of HMGB1 mRNA expression and TLR4 protein translation in sterile inflammation of mice [4]. In the sublining layer of joints in rheumatoid arthritis and osteoarthritis. Synovial macrophages exposed to HMGB1 release TNF-α, IL-1β and IL-6. HMGB1 is also present in human atherosclerotic plaques [22].

### 4.5 HMGB1 protein in brain injury

In brain injury setting, HMGB1 can be found in CSF and brain tissue. In CSF, there is no change in concentration over time. High level of HMGB1 is associated with poor outcome in severe paediatric brain injury and associated with high intracranial pressure in adults. The concentration of HMGB1 in the CSF is not associated with age or mechanism of injury. In brain tissue, HMGB1 is translocated to cytoplasm of cells in contused area at 30 min to 1 day and is localized to cytoplasm of phagocytic microglia at days 2–20. It is also found that RAGE as HMGB1 receptor, is increased in expression in contused area in phagocytic microglia [23,24]. A study had described that CSF HMGB1 is not hyperacetylated which indicates that HMGB1 is not actively secreted by neurons but rather released from damaged cells and HMGB1 might also be secreted by inflammatory cells subject to delayed activation in the postischemic brain [25].

In an in vitro trial, HMGB1 worsens excitotoxic and ischemic neuronal death and HMGB is abundantly expressed in the brain and released during cerebral ischemia. HMGB1 is induced during glia activation and triggers expression of pro-inflammatory mediators [26] in paracrine and autocrine manner [27]. This pro-inflammatory mediators will upregulate chemotaxis and chemoattractant leading to leukocyte activation as an immune response as shown by neutrophils accumulation in the brain after a brain injury. There has been shown from previous study that metalloproteinase protein (MPO) is increased in brain injury, but its correlation with HMGB1 level in yet not known [7,28,29].

Administration of HMGB1 box A fragment (as a competitive antagonist agent) inhibited brain edema by downregulation of AQP4 following brain injury. HMGB1 box A protect blood-brain-barrier (BBB) and suppressing pro-inflammatory cytokines (IL-1β, IL-6, and TNF-α), reduced cell degeneration and improved neurological performance in TBI mice [10]. Administration of *anti*-HMGB1 mAb (neutralizing *anti*-HMGB1 monoclonal antibody) in brain injury model in rat showed an association with inhibition of HMGB1 translocation, protection of BBB integrity, suppression of inflammatory molecule expression, and improvement of motor function [30].

## 5 Conclusion

In conclusion, HMGB1 has a strong pro-inflammation property which predominantly acts through RAGE pathways. This pro-inflammatory process needs to be balanced with anti-inflammatory agents for homeostasis and to provide better outcome. Further studies are needed to support anti HMGB1 therapy and role of anti-inflammatory agents in inflammation process.

## Funding sources

The article was funded privately (Authors). No sponsors have been involved.

## Ethical approval

The article is a literature review, did not require ethical approval.

## Consent

None.

## Author contribution

Thomas Tommy (TT), Mochammad Hata (MH), Andi Asadul Islam (AAI) initiated and designed the study. TT, Agussalim Bukhari (AB), MH, AAI drafted the manuscript. All authors have read and approved the final manuscript.

## Declaration of competing interest

There are no conflict of interest to be disclosed.
